# Vitamin D Status and Physical Activity during Wintertime in Forensic Inpatients—A Randomized Clinical Trial

**DOI:** 10.3390/nu13103510

**Published:** 2021-10-05

**Authors:** Anita L. Hansen, Gina Ambroziak, David M. Thornton, James C. Mundt, Rachel E. Kahn, Lisbeth Dahl, Leif Waage, Daniel Kattenbraker, Bjørn Grung

**Affiliations:** 1 Department of Psychosocial Science, University of Bergen, Christiesgt. 12, 5015 Bergen, Norway; 2 Centre for Research and Education in Forensic Psychiatry, Haukeland University Hospital, 5021 Bergen, Norway; lwaage@online.no; 3 Sand Ridge Secure Treatment Center (SRSTC), P.O. Box 0700, 1111 North Road, Mauston, WI 53948, USA; Gina.Ambroziak@dhs.wisconsin.gov (G.A.); davidsmthornton@icloud.com (D.M.T.); James.Mundt@dhs.wisconsin.gov (J.C.M.); Rachel.Kahn@dhs.wisconsin.gov (R.E.K.); Daniel.Kattenbraker@dhs.wisconsin.gov (D.K.); 4 FASTR, LLC 1213 N. Sherman Avenue, Suite 334 Madison, Madison, WI 53704, USA; 5 Institute of Marine Research (IMR), P.O. Box 1870 Nordnes, 5817 Bergen, Norway; lisbeth.dahl@hi.no; 6 Department of Chemistry, University of Bergen, Allégaten 41, 5007 Bergen, Norway; bjorn.grung@uib.no

**Keywords:** vitamin D status, physical activity, winter, supplements, randomized control trial

## Abstract

This study aimed to gain deeper knowledge about the relationship between vitamin D and physical activity in a sample of forensic inpatients. Sixty-seven male forensic inpatients participated. Participants were randomly assigned into an Intervention group (vitamin D) or a Control group (placebo). The Physical Activity–Rating (PA-R) questionnaire was used to measure physical activity from January to May. Vitamin D status was measured as 25-hydroxy vitamin D (25-OHD) pre- and post-intervention. The results revealed that vitamin D status at post-test was positively correlated with physical activity, but there was no effect of vitamin D supplementation looking at the two randomized groups. However, controlling for body mass index (BMI), the results showed an effect of BMI and a main effect of groups with a higher level of physical activity in the Intervention group. No interaction effects were found. Participants were also assigned into High and Low vitamin D groups based on the vitamin D status at post-test; i.e., the upper (75.1 nmol/L) and lower quartile (46.7 nmol/L). T-tests revealed that participants with a vitamin D status above 75 nmol/L showed significantly higher levels of physical activity than participants with a vitamin D status below 46.7 nmol/L. Thus, a vitamin D status above 75 nmol/L seems to be an optimal level.

## 1. Introduction

Recent research in a sample of forensic inpatients found that regular consumption of fatty fish such as salmon during wintertime was associated with regular physical activity, while a diet without salmon or other types of fatty fish was associated with irregular and successively declining physical activity [1]. In general, wintertime is associated with a range of negative effects on health such as mood or mental health problems (e.g., seasonal affective disorder) [2] and less physical activity along with a tendency to gain weight and higher body mass index (BMI) [3]. In one study, wintertime caused increased heart rate in a control group receiving their habitual diet in a fatty fish intervention study [4] as well as poorer stress resilience [5]. Additionally, wintertime, compared to other seasons, is associated with higher rates of mortality due to cardiovascular events. This high death rate during wintertime is related to vitamin D deficiency [6]. As wintertime seems to have so many negative health effects, and physical activity on the other hand is regarded as one of the most important resilience-enhancing strategies [7], physical activity in winter might act as an important compensatory strategy.

Low concentrations of vitamin D (e.g., 25-hydroxyvitamin D (25-(OH)D)) is a general challenge for most people during the winter, especially among people living in in high latitudes. Vitamin D varies through the year, and has its nadir in spring [8,9,10]. Many of the negative health effects during wintertime may therefore be related, in part, to this reduction in vitamin D. Vitamin deficiency is associated with a range of health problems both physical (e.g., cardiovascular diseases, cancer, bone health) and mental (e.g., schizophrenia and depression); see [8,11] for overviews. In a double blinded vitamin D study, a significant reduction in vitamin D during wintertime was associated with abnormal psychophysiological responses to an experimental stress procedure, while vitamin D supplementation during wintertime was related to normal psychophysiological responses to a stress procedure [12]. Thus, a classic nadir in vitamin D status during springtime was associated with reduced stress resilience during springtime.

Vitamin D can be regarded as a nuclear steroid hormone, and it has many physiological roles, such as regulation of calcium homeostasis [13]. Vitamin D is also important for serotonin [14], which is involved in psychological functioning (e.g., emotional regulation and cognition) and energy balance [15]. The most important sources of vitamin D are sun exposure, diet, such as fatty fish (e.g., salmon, mackerel, herring), and supplements [8]. The definition of an optimal level of vitamin D has been a matter of debate for many years. However, most researchers and experts agree that vitamin D deficiency can be defined as serum or plasma 25-hydroxyvitamin D (25-OHD) below 50 nmol/L. Vitamin D in the range of 52–72 nmol/L has been considered an insufficient level of vitamin D, while a level of 75 nmol/L or more has been regarded as a sufficient level [8,11]. During wintertime, regular consumption of fatty fish as well as vitamin D supplements may prevent a classic nadir during spring and vitamin D deficiency in general. However, it is also important to be aware that some people do not absorb vitamin D due to various reasons. Dark skin is one important factor that has been associated with lower vitamin D status, as darker skin protects against UV-radiation [16]. A lack of absorption may be related to conditions such as fat malabsorption syndromes and bariatric patients may be unable to absorb the fat-soluble vitamin D [8,11]. Indeed, an inverse relationship has been found between vitamin D and BMI > 30 kg/m^2^. In addition, research has shown that an increase in blood vitamin D3 concentrations was 57% less in obese participants compared to the non-obese participants 24 h after exposure to UV-B irradiation [17]. The relationship between low vitamin D status and obesity problems in general has been explained by a possible effect of dilution due to the body size since vitamin D status is measured by concentration in the blood e.g., [16]. Thus, it has been suggested that oral vitamin D may correct for vitamin D deficiency in obese people, but that higher doses may be required [17].

Vitamin D is positively associated with physical activity [18]. In a study by van den Heuvel [19], the authors speculated whether physical activity influenced vitamin D due to changes in hormone levels. In addition, indoor muscle-building exercise is associated with increased levels of vitamin D [20]. One explanation for this may be that vitamin D can be stored in muscles [21]. A more recent study revealed a positive relationship between vitamin D and cardiorespiratory fitness measured by maximum oxygen consumption (VO2 max) in a middle-aged US population [22]. In this study, participants were divided into the upper and lower quartile, and participants in the upper quartile for vitamin D were found to have significantly higher VO2 max compared to participants in the lower quartile. It was further emphasized that the beneficial effects of vitamin D may go beyond bone health and that further research on the effects of vitamin D are needed. It was also argued that an important research topic is to identify the optimum vitamin D status needed for cardiovascular health. As this was a cross-sectional study, it could not infer causality, and researchers were strongly encouraged to conduct randomized clinical trials investigating the effects of vitamin D supplements on cardiorespiratory fitness [22].

Thus, there seems to be an agreement in the literature that there is a relationship between physical activity and vitamin D. However, the cause of this relationship is not clearly understood. As natural exposure to sunshine is one of the most important sources of vitamin D, and most studies investigating the relationship between physical activity and vitamin D are limited to self-report, there is a risk of bias. In a randomized control trial investigating the effects of fatty fish consumption during winter on physical activity [1] it was speculated whether the beneficial effects of fatty fish consumption on physical activity could be related to vitamin D. In other words, could the reduction in vitamin D be the cause of the irregular and successive reduction in physical activity found in the control group? However, firm conclusions about the importance of vitamin D could not be made in that study [1], as fatty fish also contains other important nutrients [23].

Thus, the primary aim of this randomized clinical trial was to investigate the effects of vitamin D supplementation on physical activity during wintertime in a group of forensic inpatients. As the clinical applicability of vitamin D is not clearly understood and there are individual differences in how individuals absorb vitamin D, the secondary aim was to investigate the levels of physical activity in participants with high and low vitamin D status measured at post-test.

## 2. Materials and Methods

### 2.1. Study Design

The current study is part of a parallel randomized double-blind placebo-controlled trial that examined how vitamin D supplements effect mechanisms of stress resilience in a sample of forensic inpatients; see [12]. The study protocol was reviewed by the regulatory agencies including the Regional Committee for Medical Research Ethics, Western Norway (REK-West; 2017/1520; 6 October 2017), and the Sand Ridge Secure Treatment Center Institutional Review Board (IRB00002675; FWA00021540; 7 August 2017). The current study followed the updated Consolidated Standards of Reporting Trials (CONSORT) criteria and the trial was registered with clinicaltrials.gov (ClinicalTrials.gov Identifier: NCT03336125).

### 2.2. Study Participants

To determine the number of participants needed for the study, a priori power analysis (α = 0.05) was conducted. This analysis confirmed that 50 participants per group (n1 = n2 = 50) was needed for a Type II error level of 20%. The analysis was based on previous research [24].

**Figure 1:**
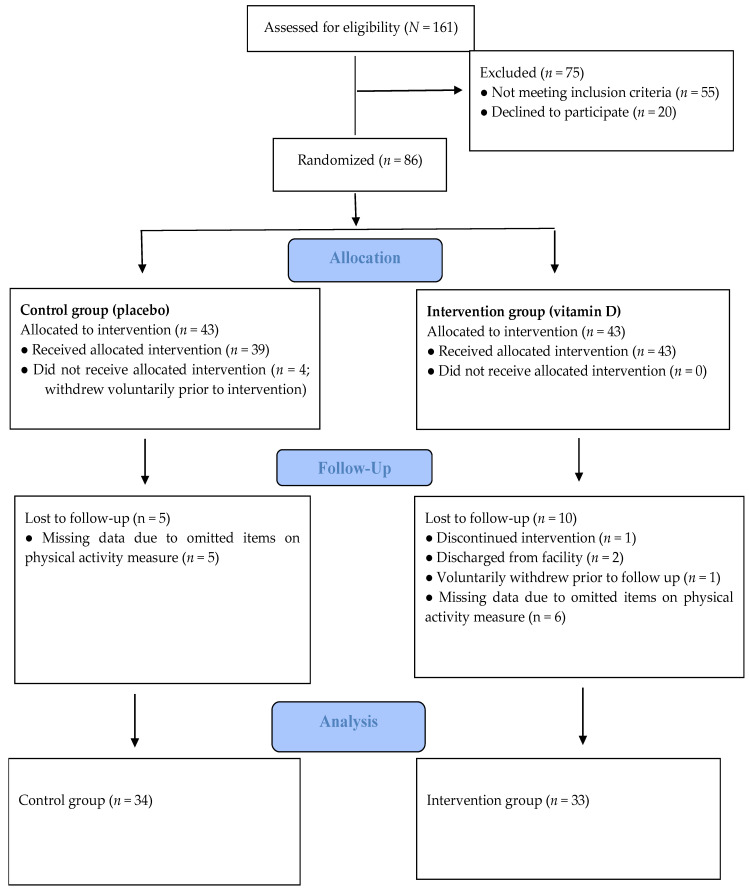
Study progress, which is in line with the CONSORT criteria.

All patients at a secure inpatient treatment facility were invited to participate in a research study on nutrients and mental health. As reported previously [12], participants were informed about the study (recruitment began 12 October 2017) using both oral and written communication. One hundred and sixty-one male patients expressed interest in participating and were assessed for eligibility. Seventy-five patients were excluded during eligibility screening. Fifty-five of these did not meet the inclusion criteria for the study and twenty patients decided against participating after receiving more information about the study. Reasons for exclusion were as follows: already taking a vitamin D supplement (*n* = 29), an IQ score of <70 (*n* = 16), presence of a severe mental illness, including schizophrenia and schizoaffective disorder (*n* = 5), presence of a major neurocognitive disorder, neurodevelopmental disorder, or history of traumatic brain injury (*n* = 2), or were unable to complete the study protocol (i.e., not fluent in English [*n* = 1]; tremor [*n* = 1]; legally blind [*n* = 1]). Thus, a total of 86 volunteers were randomized equally into two groups of 43: Control group (placebo) and Intervention group (vitamin D). On the Physical Activity –Rating (PA-R) questionnaire we had missing data on 11 participants due to some omitted items. Thus, we had physical activity data on 67 participants. A flow diagram of the study process is presented in Figure 1. Mean (SD) age for the current study sample (*N* = 67) was 49 (12) years (range 31–81).

A detailed description of the randomization procedure, which was in line with the CONSORT criteria, was published previously [12]. Before participating in the study, participants were informed of their rights and signed an informed consent document. Participants were told they had the right to withdraw from the study at any time without penalty and participation in the study would not have any effect on their confinement or treatment status as a patient in the institution.

### 2.3. Intervention

The intervention period started 7 January 2018 and ended 22 May 2018. The Intervention group received vitamin D pearls daily (40 µg cholecalciferol corresponding to 1600 IU), while the Control group received placebo pearls (120 mg olive oil). These were provided as identical clear round soft gelatin pearls containing a light brown oil. Along with their daily medication, participants had the supplements delivered to their unit daily. Health Services staff delivered the supplements and tracked compliance with taking the supplements by recording a “1” if the participants took the capsules and “0” if the participant did not take the capsules. In the event that a participant left the institution temporarily (e.g., out to a medical appointment or court hearing) the supplements were packaged along with his other medication to be administered off-site. As it was reported in Hansen et al. [12] the compliance was high, at about 97%.

The amount of vitamin D supplied was based on recommendations from the National Institute of Health (NIH). The current American and Nordic upper limit for vitamin D intake is 100 µg/day (4000 IU/day). This is the highest daily intake level recommended that is likely to pose no risk of adverse health effects to the general population [25]. The vitamin D pearls in the present study contributed 38% of the upper limit intake level. The vitamin D pearls were produced by Pharma Nord, Denmark and were Halal and Kosher certified.

As described previously [12], participants were offered up to USD 40 in compensation for participating in the study. Providing compensation to voluntary participants is a common practice in the U.S. For completing the pre-intervention test battery, participants were paid USD 10. All participants were required to maintain compliance with the supplement (or placebo) regimen throughout the study. Participants who missed more than three doses of supplements during any one week of the intervention period were no longer eligible to continue in the study. Participants who maintained compliance with supplement intake and completing the post-intervention test battery were compensated with an additional USD 30 upon completion of the study.

### 2.4. Measures

The Physical Activity–Rating (PA-R) questionnaire was used to measure level of physical activity. This questionnaire is also known as the Physical Activity Status Scale [26], and University of Houston Non-Exercise Test for estimation of maximum oxygen consumption [27]. This instrument was developed by the National Aeronautics and Space Administration’s (NASA) Johnson Space Center [28]. The questionnaire contains three main categories, which are divided into seven subgroups. Listed in order of increasing activity level, the main categories, subgroups and their codes are as shown in Table 1. 

Thus, from the start of the intervention period (7 January 2018) to the end of the intervention (22 May 2018) the participants completed the form on a weekly basis. They recorded the frequency, duration, and type of physical activity by filling in the respective code in the form. For statistical analyses, average scores from each month were used. An average score for the whole intervention period was also computed.

#### Blood Sampling

As described previously [12], fasting blood was collected from participants in the morning between 0630 and 0930 by biomedical health services staff at the treatment facility pre- and post-intervention. Pre-test was carried out from mid-November to mid-December and post-test was carried out from the beginning of April to mid-May. Venous blood for serum preparation was collected in BD Vacutainer^®^ vials and set to coagulate for a minimum of 30 min before centrifuging (1000–1300 G, 20 °C, 10 min) within 60 min. Samples were transported the same day to a clinical laboratory near the study site (ACL laboratories, Milwaukee, WI, USA) for analysis of vitamin D (i.e., 25(OH)D). For the determination of vitamin D, a competitive immunoassay was used and a vitamin D analog labeled with fluorescein for detection. The unit of measurement for 25(OH)D was nanomole per liter (nmol/L).

### 2.5. Statistical Analysis

To investigate the overall relationship between vitamin D status and physical activity during the intervention period Pearson Product Moment correlation analysis was used. Correlation coefficients are reported as *r*. Repeated Measures Analysis of Variance (ANOVA) was used to determine whether vitamin D supplements had any effects on the level of physical activity during wintertime. Intervention and Control groups were treated as independent variables and physical activity (average scores per month) was the dependent variable. For these analyses *F*-values are reported to illuminate whether there is a statistical difference between two means. Moreover, we also performed analyses controlling for BMI [17]. Significant effects (*p* < 0.05) were followed up with Fisher’s Least Significant difference (LSD) test.

To gain deeper knowledge about the clinical applicability of the vitamin D status, participants were also assigned into High and Low vitamin D groups based on the vitamin D status at post-test; i.e., the upper (75.1 nmol/L) and lower quartile (46.7 nmol/L) of the post-vitamin D status measure. This was done to exclude participants in the borderline area and provide an even clearer picture of the functional role of a vitamin D level of 75 nmol/L or more which is regarded as a sufficient level [8,11]. *t*-tests were used to test the differences between the two groups. For significant differences effect sizes Cohen’s *d* was calculated to determine the magnitude of the differences [29].

## 3. Results

### 3.1. Descriptive Statistics

Descriptive statistics from this randomized control trial (RCT) have been reported previously [12]. However, means and standard deviations for relevant descriptive variables for the current sample included in this study (*N* = 67) are presented in Table 2.

### 3.2. Correlations

No significant correlations were found between vitamin D status measured at pre-test and physical activity. However, vitamin D status measured at post-test correlated significantly with physical activity performed during the whole intervention period; January (*r* = 0.38, *p* = 0.001), February (*r* = 0.33, *p* = 0.004), March (*r* = 0.31, *p* = 0.007), April (*r* = 0.29, *p* = 0.010) and May (*r* = 0.27, *p* = 0.001), and average score for the whole intervention period (*r* = 0.40, *p* = 0.001).

BMI was significantly negatively correlated with physical activity throughout the whole intervention; January (*r* = −0.25. *p* = 0.044), February (*r* = −0.26, *p* = 0.033), March (*r* = −0.24, *p* = 0.052), April (*r* = −0.26, *p* = 0.036) and May (*r* = −0.25, *p* = 0.043).

### 3.3. Effects of Vitamin D Supplementation during Winter on Physical Activity

Repeated measures analysis of variance revealed no effects of groups, *F*(1,65) = 2.09, *p* = 0.153. Nor were there any effects of time, *F*(4,260) = 2.10, *p* = 0.081 or interaction effects *F*(4,260) = 0.09, *p* = 0.986 (see Table 3).

Controlling for BMI, the results revealed that there was a significant effect of BMI, *F*(1,64) = 5.11, *p* = 0.027. However, no effect of time *F*(1,256) = 0.21, *p* = 0.931, or any interaction effects between time and BMI *F*(4,256) = 0.07, *p* = 0.991, or time and groups *F*(4,256) = 0.09, *p* = 0.987 were found.

Excluding the four participants in the Intervention group who did not increase in vitamin D status from the data analysis, the repeated measures analysis of variance revealed a marginal effect of groups, *F*(1,61) = 3.82 *p* = 0.055, which can be considered a trend. Follow-up tests showed the level of physical activity in the Intervention group was higher compared to the Control group, but this difference was not statistically significant (*p* = 0.055). There was no effect of time, *F*(4,244) = 1.93, *p* = 0.106, nor any interaction effect between time and groups, *F*(4,244) = 0.16, *p* = 0.957 (see Table 2).

When controlling for BMI, in addition to the exclusion of four participants, there was a significant effect of BMI, *F*(1,60) = 4.66, *p* = 0.035. There was also a significant effect of groups, *F*(1,60) = 4.16, *p* = 0.046. Follow-up tests showed the Intervention group had a significantly higher level of physical activity compared to the Control group (*p* = 0.046). No effect of time, *F*(4,240) = 0.23, *p* = 0.924, or any interaction effects were found between time and BMI, *F*(4,240) = 0.07, *p* = 0.990, or time and groups, *F*(4.240) = 0.16, *p* = 0.959.

### 3.4. Differences between High and Low Vitamin D Status in Physical Activity

When participants were assigned into groups on the basis of the upper and lower quartile, *t*-tests revealed that participants with higher vitamin D status at post-test showed higher levels of physical activity compared to participants with lower vitamin D status throughout the whole intervention period (see Table 4).

## 4. Discussion

The results from the present study revealed that vitamin D status at post-test correlated significantly with physical activity throughout the whole intervention period, but the current study revealed no overall effect of vitamin D supplementation when the Intervention and the Control group were treated as independent variables. After controlling for BMI, the results revealed a main effect of BMI as well as a main effect of groups showing that the vitamin D group had demonstrated greater levels of physical activity compared to the placebo group. Moreover, when participants were assigned into Low and High vitamin D groups based on the vitamin D status at post-test (i.e., upper vs. lower quartile), the results showed that participants with higher vitamin D status (i.e., >75 nmol/L) had higher levels of physical activity than participants with low vitamin D status.

The positive association between vitamin D status and physical activity is in line with previous research [18], although this does not imply causation. The current study expanded on past research using a randomized clinical trial to investigate the effects of vitamin D supplements on physical activity during wintertime. Overall, the repeated measures ANOVA revealed no effect of vitamin D supplementation. To gain deeper insight into the present results, the vitamin D levels of individuals within the two randomized groups was explored. Table 1 shows the vitamin D status for the sample in this study. The vitamin D status for the Intervention group increased significantly from pre- to post-test. The mean level of vitamin D for the Intervention group was 64.3(16.9) nmol/L at pre-test, and it increased to 75.4(21.9) nmol/L at post-test, which is slightly above the recommended level of 75 e.g., [8]. The Control group had a vitamin D level of 57.7(21.5) nmol/L at pre-test and it decreased to 46.1(15.3) nmol/L at post-test. Importantly, these numbers are *means at group level*. Even if there was a significant increase in vitamin D level in the Intervention group overall, not all of the participants in the Intervention group reached a post-test vitamin D status regarded as sufficient according to US recommendations, i.e., 75 nmol/L, e.g., [8].

A closer look at individual values revealed that four participants in the Intervention group did not improve their vitamin D status from pre- to post-test. Similarly, three of the participants in the Control group had high levels of vitamin D (above 75 nmol/L) at post-test. These control participants also had a very high vitamin D status at pre-test. At pre-test the vitamin D status ranged between 31.0 nmol/L and 91.9 nmol/L for the Intervention group, and between 20.2 nmol/L and 102.3 nmol/L for the Control group. At post-test the range was 37.2–125.5 nmol/L for the Intervention group, and 24.5–84.1 nmol/L for the Control group. Thus, the high vitamin D status during pre-test in some of the control participants seemed to prevent a classic nadir in vitamin D during spring. As patients were excluded from participation in the study if they were already taking vitamin D supplements, the reason for this high vitamin D level at pre-test might be due to sun-exposure during the late summer. While the patient access to outdoor activity is restricted, they still have substantial opportunity to be outdoors. There are a few structured options for outdoor recreation each day at scheduled times. In addition, at other times of the day (except overnight or during formal institution counts or other security protocols), patients can go into an outdoor “courtyard” attached to their housing units. These courtyards are small, mostly cement/concrete areas, but they are outdoors with direct sunlight. This means the amount of time the participants spent outside could vary considerably with some getting little to none and others getting a couple hours each day. However, an important question is why the participants in the Intervention group did not increase in vitamin D status from pre- to post-test, given the high level of compliance (97%) in this study [12]. One explanation to consider is that some individuals do not absorb the vitamin D supplements due to individual differences such as high BMI and the effect of dilution [e.g., 16, 17]. Controlling for BMI in the current study also revealed a significant effect of BMI on physical activity. In addition, correlational analyses showed a negative association between BMI and physical activity. Importantly, individuals show individualized responses to vitamin D and can be segregated into high, mid, and low responders to supplementation of vitamin D. Two RCT studies from Finland indicated that approximately 25% of the participants were low responders and needed to take higher daily vitamin D doses (maybe 50–100 µg) than high responders (10–20 µg) to achieve a sufficient level of vitamin D see [30] for an overview. Another explanation, although unlikely, could also be that these four participants did not take the supplements as directed.

When groups were formed on the basis of upper and lower quartiles, the results revealed that participants in the High vitamin D group had significantly higher levels of physical activity compared to those in the Low vitamin D group. These results support and expand the results from Marawan et al. [22], as the present results suggest that a level of 75 nmol/L or more is associated with regular physical activity over time (from January to May). Throughout the whole intervention period the level of physical activity performed by the High vitamin D group is in line with Category 3 of the PA-R (i.e., regularly more difficult physical activity such as running or jogging, spinning or other forms of aerobic training). In contrast, the level of physical activity performed by the Low vitamin D group is in line with Category 2 of the PA-R (i.e., moderate physical activity such as jogging, ping pong/table tennis, resistance training, or hard physical work). The current results are similar to results found by Hansen et al., [1]. In that study, participants in a fatty fish group could be categorized in the Category 3 of the PA-R as well, while the control group was closer to the Category 2, especially during the last five weeks of the six months intervention period. However, when comparing the mean physical activity score for the whole intervention period with the overall mean physical activity score in Hansen et al. [1], the present study illuminates a larger distinction or gap between High and Low vitamin D groups than the fatty fish and control groups did [1]. The mean physical activity score for the High vitamin D group was 4.63 in the current study, while the mean physical activity score for the Low vitamin D group was 2.50. In Hansen et al. [1] the mean physical activity score for the fatty fish group was 4.04 and for the control group it was 3.50. Thus, the present study supports and expands earlier studies [1,22] showing that vitamin D seems to be important for levels of physical activity throughout the winter, with a vitamin D level above 75 nmol/L being optimal.

The present study has some limitations to consider. The sample size is small, and thus conclusions should be made with caution. As the present study includes only male forensic inpatients, we do not yet know whether it is possible to generalize these results to other populations, such as the elderly, females or trans individuals. Thus, future research should investigate the effects of vitamin D during winter on physical activity in other populations and within larger samples. The present study also did not include an objective measure of physical activity such as oxygen consumption. However, the present study also has some important strengths worth highlighting. The intervention in this study was carried out during winter in a sample with restricted access to outdoor activity compared to other populations. Importantly, travelling to more sunny places which is very common during wintertime in other populations, was not an option for the participants in this study. Moreover, the intervention period lasted for a longer period of time compared to many other supplement intervention studies and post-testing took place while the participants were still taking supplements to avoid a possible decline in vitamin D after supplement termination. Importantly, the current study was a randomized control trial, with a high level of compliance.

## 5. Conclusions

The present study expands the clinical applicability and significance of vitamin D. Vitamin D status seems to have implications for levels of physical activity, one of the most resilience enhancing strategies for both physical and mental health. Thus, results from the present study may have important clinical implications for vulnerable individuals with both mental and physical health problems. Although the present study design was a randomized control trial, many factors may influence the levels of physical activity during wintertime and the cause-and-effect relationship between physical activity and vitamin D is still not clearly understood. Thus, future randomized control trials must include a larger sample size. However, the present study indicates that a vitamin D status above 75 nmol/L is optimum level to maintain physical activity within the Category 3 of the PA-R during wintertime. In turn, maintaining this level of physical activity (i.e., physical exercise) has been shown to be important for both physical and mental health [31].

## Figures and Tables

**Table 1:** Shows the different categories in the Physical Activity–Rating (PA-R) questionnaire.

Category 1 ^a^	Category 2 ^b^	Category 3 ^c^
0 = No physical activity at all	2 = 10–60 min per week	4 = Running, spinning or other such activity less than 30 min per week
1 = Little physical activity, such as going for a walk	3 = More than 1 h per week	5 = Running, spinning or other such activity for 3–5 miles per week or about 1 h per week
		6 = Running, spinning or other such activity for 5–10 miles per week or between 1 to 3 h per week
		7 = Running, spinning or other such activity more than 10 miles per week or more than 3 h per week

Note: ^a^ Participation in little or no physical activity, ^b^ Participation in physical activity or work that requires moderate physical activity, such as jogging, ping pong/table tennis, resistance training, or hard physical work, ^c^ Participation in regular hard physical activity such as running or jogging (treadmill), spinning or other forms of aerobic training [26,27,28].

**Table 2:** Means and standard deviations for descriptive variables.

Descriptive Variables	Intervention Group (*N* = 33)	Control Group (*N* = 34)
Age	48.2(12.1)	49.5(12)
BMI ^a^	30.9(7.5)	30.6(7.6)
25(OH)D concentration (nmol/L) ^b^		
Pre-test	64.3(16.9) Range: 31.0–91.9	57.7(21.5) Range: 20.2–102.3
Post-test ^c^	75.4(21.9) Range: 37.2–125.5	46.1(15.3) Range: 24.5–84.1

^a^ The BMI measure presented in the table was taken at pre-test. BMI did not change from pre- to post-test. ^b^ Repeated measures of analysis for the current sample (i.e., *N* = 67) showed that there were no between-group differences in vitamin D status at pre-test (*p* = 0.16), but there was a between-group difference at post-test (*p* < 0.001). Within-group analyses showed that the Intervention group increased significantly in vitamin D status from pre- to post-test (*p* < 0.001), while the Control group had a significant decrease (*p* < 0.001). These results are in line with the results from the whole sample (*N* = 78) [12]. ^c^ The range in vitamin D for the Intervention group was 37.2–124.5 nmol/L. Given the lowest vitamin D level was below 50 nmol, which is regarded as a vitamin D deficiency, a closer examination of individual values was conducted. Four participants in the Intervention group had a vitamin D level at post-test below 50 nmol. These participant’s vitamin D status did not increase from pre- to post-test. Three participants in the Control group had a vitamin D level above 75 nmol/L.

**Table 3:** Means and standard deviations for physical activity (average per month) throughout the intervention period for the Intervention and the Control groups.

**Physical Activity**	**Intervention Group (*N* = 33)**	**Control** **Group (*N* = 34)**
January	3.6(2.3)	2.9(2.0)
February	3.9(2.3)	3.1(2.1)
March	3.7(2.1)	3.0(2.2)
April	3.8(2.2)	3.1(2.2)
May	4.0(2.1)	3.3(2.2)
^a^	**Intervention** **Group** **(*N* = 29)**	
January	3.86(2.27)	
February	4.17(2.22)	
March	4.16(2.08)	
April	4.05(2.19)	
May	4.23(2.10)	

Note: ^a^ This part of the table shows the recalculated means and standard deviations for the Intervention group leaving out the four participants that did not increase in vitamin D level from pre- to post-test (*N* = 29).

**Table 4:** Significant differences between High (upper quartile, i.e., >75.1 nmol/L) and Low (lower quartile, i.e., <46.7) vitamin D groups on average physical activity per month from January to May.

Physical Activity	Low Vitamin D	High Vitamin D	*t*-Value	df	*p*	*d*
	Range: 24. 5–46.7	Range: 75.1–125.5				
January	2.4(1.7) (N = 19)	4.6(2.1) (N = 17)	−3.5	34	0.001	1.16
February	2.7(1.8) (N = 20)	4.3(2.3) (N = 20)	−2.35	38	0.024	0.74
March	2.6(1.7) (N = 20)	4.4(2.1) (N = 20)	−2.9	38	0.006	0.92
April	2.5(1.6) (N = 20)	4.2(2.2) (N = 20)	−2.91	38	0.006	0.92
May	2.5(1.5) (N = 20)	4.4(2.2) (N = 19)	−3.09	37	0.004	0.98
Average whole period	2.5(1.6) (N = 19)	4.6(2.1) (N = 16)	−3.50	33	0.001	1.17

Note: df = degrees of freedom. *d =* effect sizes; Cohen’s *d*.
